# Publicity and Public Health

**Published:** 1921-08-13

**Authors:** 


### PUBLICITY AND PUBLIC HEALTH.

A correspondent who visited the Congress of the Royal Sanitary Institute, recently held at Folkestone, was disappointed to see such a small number of the general public at the lectures and exhibition. He did not realise to how great an extent it is believed that congresses in general are meetings for experts only, and that people do not realise that public health, unlike other subjects on which conferences are called, is a subject of which we all know something. That something may be but small and inaccurate, but everyone has ideas which may be enlarged and corrected by listening to the words of the experts. For public health cannot be achieved by the action of "authorities" without the co-operation of every citizen. A leaky drain-pipe may endanger the health of many, and neglect to call in the doctor for some apparently slight ailment, which ultimately proves to be an infectious disease, may be responsible for an epidemic. In this connection the suggestion is made that when a public health conference is to be held in any town all workers in the cause of sanitation should be enlisted as voluntary publicity agents. Sanitary inspectors, district nurses, Poor-law officials might be asked to mention the fact of the Congress to those with whom they come into contact. These officials might even be provided with invitation cards to meetings of special interest to the people among whom they work. If these were issued with a blank in which the nurse, or whoever gives the card, could fill up the name of Mrs. So-and-so, some at least might go to the meetings. Even if only a few availed themselves of the invitation these few would act as missionaries among their friends. It would be necessary, of course, to have lecturers who have the popular gift, but this is by no means a matter impossible to achieve, and since things said on a platform carry more weight than things said in private conversation, a gradually increasing appreciation of the importance of health measures and the share which each of us may have in carrying them out would very probably result, with advantage to the whole nation.

